# Refractory Heart Failure of Unknown Etiology May Be Cardiac Amyloid If Preceded by Hereditary Neurological Symptoms

**DOI:** 10.7759/cureus.9392

**Published:** 2020-07-25

**Authors:** Tuoyo 0 Mene-Afejuku, Adedoyin A Akinlonu, Adenike N Graham, Ola Akinboboye

**Affiliations:** 1 Epidemiology, Rollins School of Public Health, Emory University, Atlanta, USA; 2 Cardiology, Queen’s Heart Institute, Laurelton Heart Specialist, New York, USA

**Keywords:** cardiac amyloidosis, heart failure, neuropathy, hand tremors

## Abstract

Transthyretin cardiac amyloidosis results from the deposition of transthyretin amyloid fibrils in the myocardium. This happens because of the misfolding of genetically normal (wild type - ATTR) or mutant (hereditary ATTR) transthyretin. The clinical presentation of hereditary ATTR cardiac amyloidosis is dependent on the exact site of the amino acid substitution. The V122I gene mutation is most common among people of African descent and usually manifests with cardiomyopathy. The mutations are transmitted in an autosomal dominant manner with variable penetrance and associated with clinical features occurring most commonly after the age of 40. The symptoms of heart failure (HF) may be preceded by several years of vague neurological symptoms which is more concerning if there is no clear explanation. A high index of suspicion is therefore crucial in ensuring prompt diagnosis and therapy, as this may favorably alter the gloomy prognosis associated with cardiac amyloidosis.

## Introduction

Hereditary ATTR amyloidosis, if not diagnosed early, has a high mortality rate [1,2]. The most prevalent mutation in the United States is V122I and it usually manifests with a cardiac phenotype [3]. However, many patients have neurological manifestations that often precede symptoms of heart failure (HF). A temporal profile of neurologic symptoms followed by HF symptoms as well as a family history of HF should raise the suspicion of cardiac amyloidosis [1,2]. Newer therapies have emerged which may improve the prognosis of these patients if early diagnosis is made, as hereditary ATTR amyloidosis does not seem to be as rare as previously envisaged.

Therefore, we present this case series, to highlight the significance of high index of suspicion, prompt diagnosis, genetic testing, and therapeutic modalities for this grave disease which may now be amenable to therapy.

## Case presentation

Case 1

A 51-year-old Afro-Caribbean male with a history of high blood pressure and type 2 diabetes mellitus presented to our cardiology clinic in 2015 following referral by his primary care provider for continued care. The patient had a protracted history of peripheral neuropathy, bilateral hand tremors, leg tingling/numbness, and intermittent claudication of unknown etiology. In 2014, during a brief hospitalization at another facility, he was diagnosed with congestive HF and consequently started on guideline-directed medical therapy (GDMT). The patient’s HF symptoms were refractory to therapy and the etiology of his HF was unknown.

**Figure 1:**
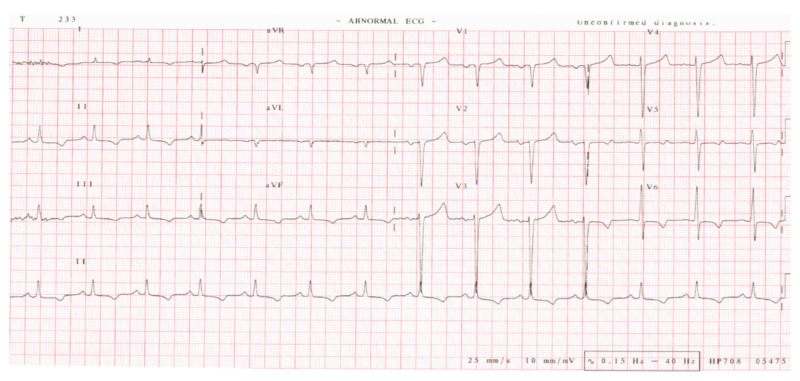
Electrocardiogram of case 1.

When he was seen at our clinic, he was still complaining of shortness of breath, chest pain, palpitations, worsening pedal swelling, worsening bilateral hand tremors, and lower limb tingling/numbness. Over a series of office visits, the patient’s physical exam continued to show significant leg swelling without evidence of hepatic disease nor venous insufficiency. A two-dimensional (2-D) echocardiogram at his index visit at our facility was notable for increased left ventricular wall thickness, left ventricular ejection fraction of 40% to 45%, global hypokinesis, biatrial enlargement moderate mitral regurgitation, and grade III diastolic dysfunction. Electrocardiogram (EKG) revealed T wave inversions in the inferolateral leads and poor R-wave progression as shown in Figure 1. The nuclear stress test revealed normal myocardial perfusion. Venous duplex showed no evidence of deep venous thrombosis. He also had a Quantitative Sudomotor Axon Reflex Test (QSART) because of his protracted history of bilateral hand tremors and tingling in the limbs. QSART revealed significant autonomic deficits in all distal extremities.

Pertinent laboratory findings are: mild microcytic anemia with stage III chronic kidney disease (serum creatinine of 2.64 mg/dL and estimated glomerular filtration rate of 32 ml/min) and hemoglobin A1C was 7.8%.

The constellation of findings of progressive and refractory congestive HF, echocardiographic features (restrictive cardiomyopathy), sudomotor results, and preceding history of long-term familial neurological symptoms and HF, triggered suspicion of cardiac amyloidosis. Genetic testing after pretest counseling revealed the presence of V122I gene mutation (heterozygote). He was also tested for the presence of clonal light chains (AL) which was negative.

He was continued on the following medications: carvedilol, frusemide, lisinopril, rosuvastatin, metformin, and insulin. He was started on tafamidis and appeared to be making good clinical progress. He was last seen in the clinic in January 2020 and at that time, his symptoms had markedly improved, and he has not been admitted for decompensated HF since he was started on tafamidis. Genetic screening was done for as many first-degree relatives as we could reach.

Case 2

**Figure 2:**
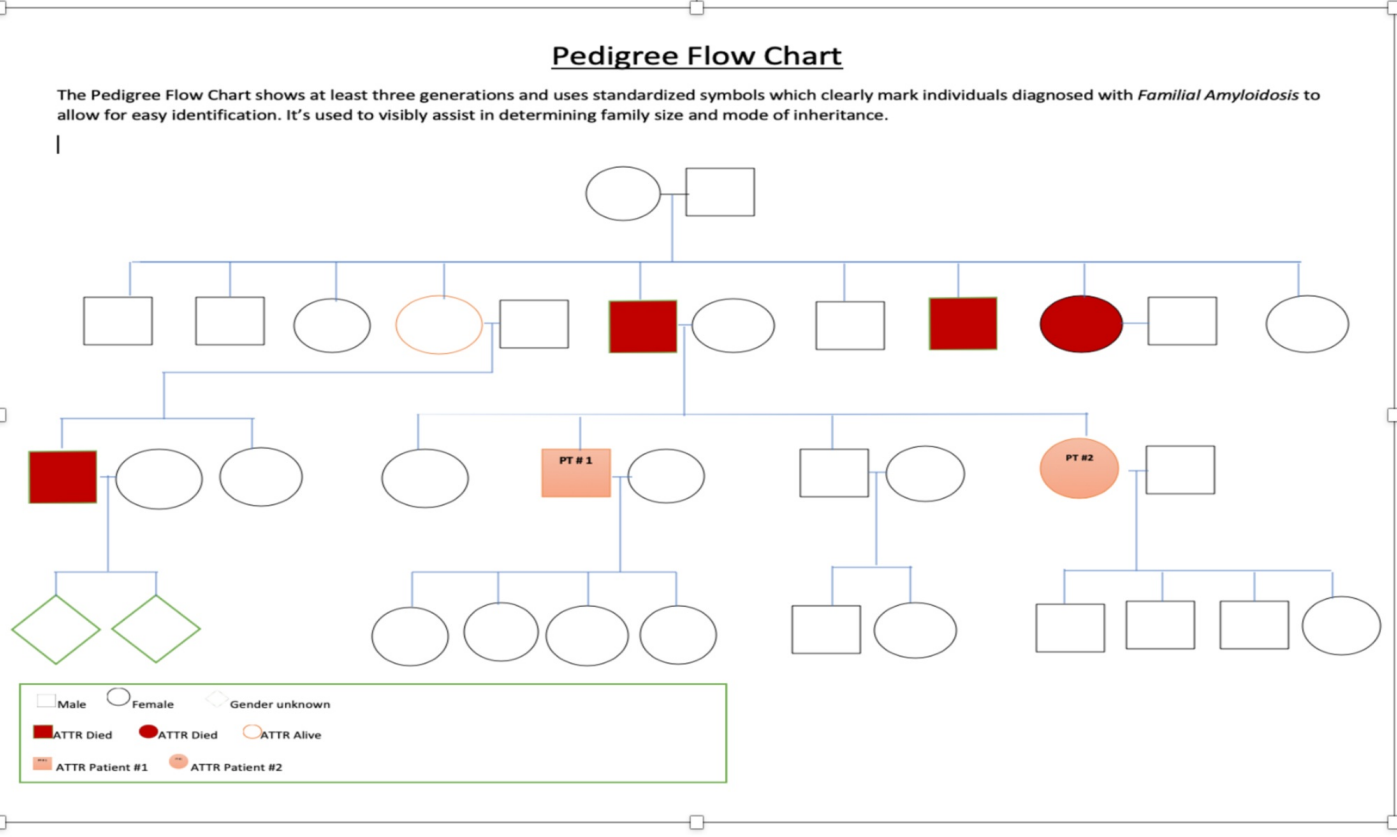
Family tree of cardiac amyloidosis in this case series.

**Figure 3:**
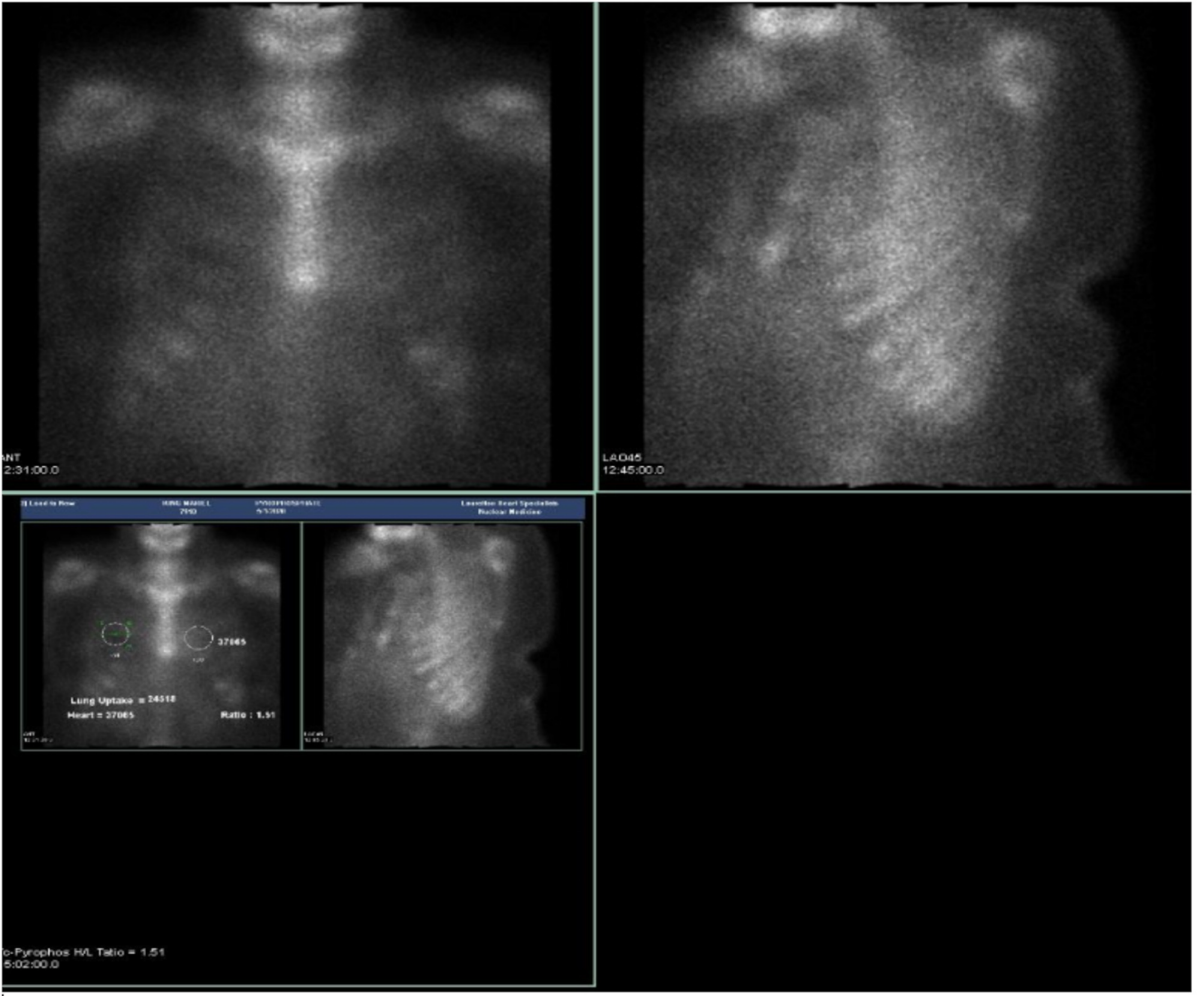
Technetium pyrophosphate scan showing a heart/lung tracer ratio of 1.51

**Figure 4:**
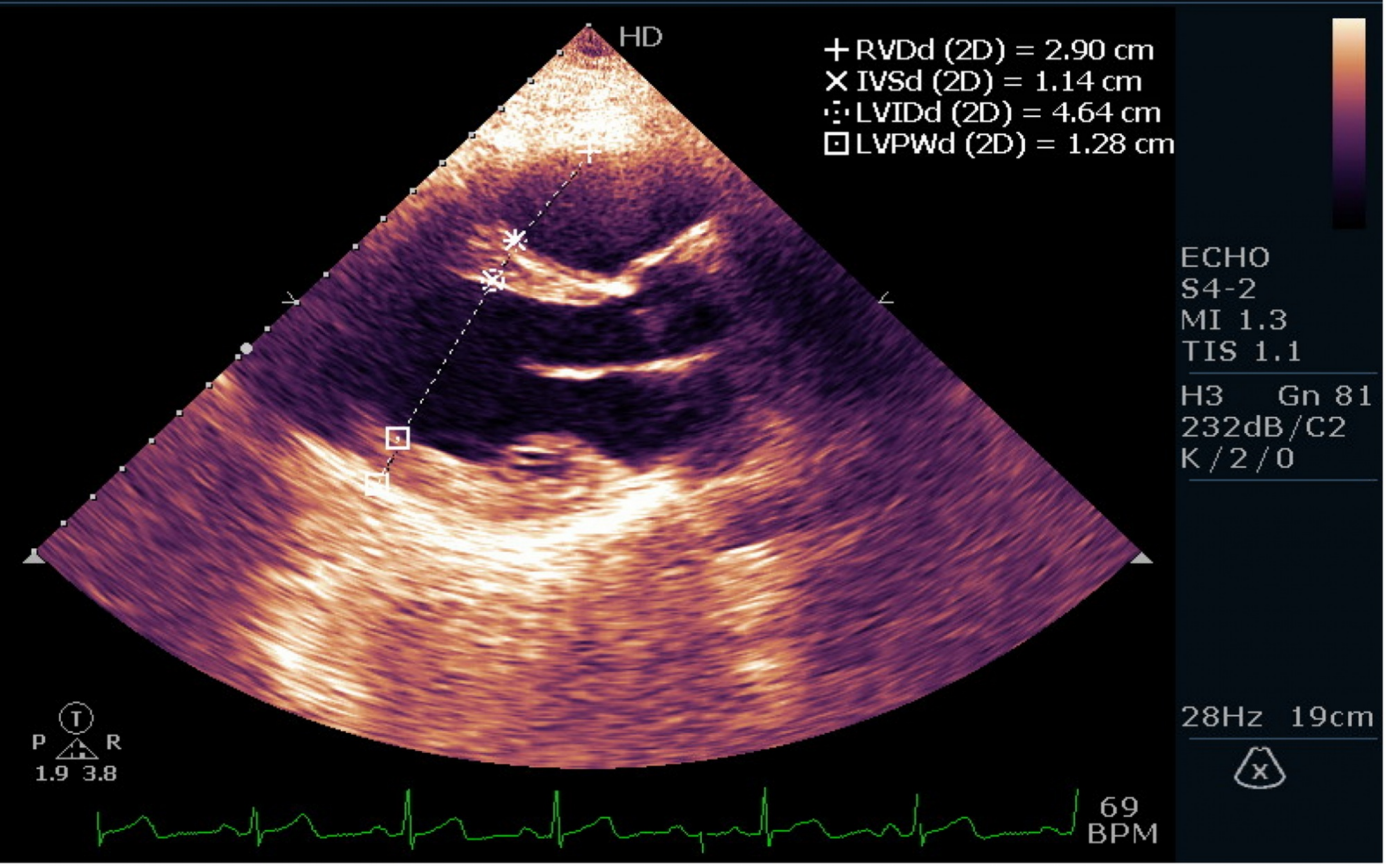
Echocardiogram for case 2.

A 40-year-old African American female (sister to case 1) presented to the cardiology clinic on account of worsening dyspnea on exertion for about two months. These symptoms were preceded by intermittent bilateral hand tremors since the age of 16 years. These hand tremors were familial and of unknown cause. The rest of the systemic review was non-contributory apart from the history of pre-diabetes. She was not on any medications at the time of her initial visit. The patient reported a history of cardiac amyloidosis in multiple family members including her father and brother who was the first case presentation (a family tree is displayed in Figure 2 below). The physical exam showed obese female but was otherwise unremarkable. The initial laboratory work-up was unrevealing. Her EKG showed normal sinus rhythm with poor R-wave progression. Technetium pyrophosphate scintigraphy scan showed a heart to contralateral lung ratio of 1.51 which is marginally higher than the cutoff of 1.5 (Figure 3). This may imply less severe disease because the condition is being detected in the early phase. Echocardiography was also unremarkable apart from mild left ventricular wall thickening (Figure 4).

Our tentative diagnosis was possible early symptoms of HF secondary to hereditary ATTR amyloidosis because of her family history. The patient had testing after appropriate genetic counseling, which revealed heterozygosity for the V122I transthyretin protein with a second gene mutation (TTN) similar to the one observed in her elder brother. Her serum-free light chain ratio was 17.2 (normal range reported by the laboratory was 0.32 to 18.6). Neurological evaluation revealed evidence of lower extremity motor neuropathy. The patient was started on treatment with weekly inotersen (Tegsedi), which she has tolerated to date. As at the last clinic visit, initial symptoms of shortness of breath had improved.

## Discussion

Patients with hereditary ATTR secondary to V122I mutation usually present with a cardiomyopathy phenotype [1-3]. However, neurological manifestations are not uncommon. These neurological symptoms cover a broad spectrum ranging from carpal tunnel syndrome, autonomic neuropathy to varying forms of peripheral neuropathy [4,5].

There are not many reports about familial bilateral hand tremors preceding symptoms of HF as in this case series. In fact, the first case was thought to have Parkinson’s disease/essential tremors which underscore the importance of good family history and strong index of suspicion especially for individuals with non-ischemic cardiomyopathy and HF symptoms refractory to standard therapy.

Upon making a diagnosis of ATTR cardiac amyloidosis, the next line of action is prompt therapy. Most of the medications for cardiac amyloidosis are novel. Tafamidis was started for the first case because he had symptomatic HF with New York Heart Association (NYHA) class 2 symptoms. Tafamidis is approved by the United States Food and Drug Administration (FDA) for cardiomyopathy. The mechanism of action is the stabilization of the TTR tetramers [6].

On the other hand, inotersen (Tegsedi) was the medication started for the second case mainly because she had prominent features of longstanding peripheral neuropathy without prominent cardiac involvement. Inotersen (Tegsedi) is a TTR silencer that inhibits hepatic TTR production [6]. As stated earlier, most of these medications are novel but showed promise in improving outcomes when compared to placebo [3,6-8]. Newer therapies are still being studied. The efficacy of some of these medications was tested on certain mutation variants and not much has been done specifically for the V122I mutation.

Apart from clinical symptoms, cardiac magnetic resonance imaging (MRI) at baseline and on follow-up may have useful prognostic implications [9]. The presence of transmural late gadolinium enhancement (LGE) is associated with more than a five-fold increase in death compared to cardiac amyloid patients without LGE. Widespread use of cardiac MRI may be expensive with the limitation of the need for sophisticated techniques to actually quantify LGE [9,10]. Renal dysfunction just like in case 1 also limits the use of cardiac MRI.

Further studies would still be needed to assess the long-term efficacy of these new therapies in terms of mortality and morbidity associated with HF. Also, targeted therapies of ATTR disease secondary to the V122I would be useful as the prevalence of this subtype appears to be more prevalent than previously envisaged. Ongoing clinical trials may elucidate more on the efficacy of medications on the V122I mutations in comparison to previous studies on the efficacy of these medications on other mutant types.

## Conclusions

Patients with hereditary ATTR amyloidosis secondary to V122I mutation usually present with cardiomyopathy. The symptoms of cardiomyopathy may be predated by subtle neurological symptoms like tremors and autonomic neuropathy as outlined in the two cases above. A high index of suspicion is required to ensure early diagnosis and institution of therapy.
